# Beyond body mass index: visceral adiposity and metabolic alterations as early markers of atrial dysfunction and atrial fibrillation in midlife

**DOI:** 10.3389/fendo.2026.1775534

**Published:** 2026-03-04

**Authors:** Angelina Borizanova, Elena Kinova, Natalia Spasova, Assen Goudev

**Affiliations:** 1 Department of Emergency Medicine, Medical University Sofia, Sofia, Bulgaria; 2 Clinic of Cardiology, University hospital (UMHAT) “Tsaritsa Yoanna- ISUL“, Sofia, Bulgaria

**Keywords:** atrial fibrillation, cardio-renal-metabolic syndrome, left atrial contractile strain, left atrial reservoir strain, obesity

## Abstract

**Background:**

Atrial fibrillation (AF) develops along the cardiometabolic continuum, where visceral adiposity and early atrial dysfunction may precede overt disease. We aimed to identify independent predictors of new- onset and recurrent AF in middle-aged individuals with cardiometabolic risk.

**Methods:**

This observational cohort included 240 outpatients (40–60 years; 71 controls, 77 new-onset AF, 92 recurrent AF). Conventional anthropometric parameters (body mass index, body roundness index) and biochemical markers (fasting glucose, uric acid, creatinine clearance, inflammatory markers and high-sensitivity troponin I- hsTnI) were assessed. A comprehensive echocardiographic assessment including left atrial reservoir strain (LASr), electromechanical delay (EMD), and epicardial adipose tissue (EAT) were analyzed. Binary logistic regression and ROC analyses were performed.

**Results:**

New-onset AF was independently associated with fasting glucose (OR 3.604; 95% CI 1.338–9.704; p=0.011), EAT thickness (OR 1.479; p=0.006), electromechanical delay (OR 1.043; p=0.001), uric acid (OR 1.006; p=0.026), and lower LASr (OR 0.944; p=0.045). Among the evaluated parameters, EAT demonstrated the highest discriminatory ability for new-onset AF (AUC 0.664; p<0.001). Recurrent AF was independently associated with age (OR 1.122; p<0.001), BMI (OR 1.209; p=0.028), hsTnI (OR 3.546; p<0.001), and lower LASr (OR 0.845; p<0.001). LASr showed good discriminatory performance for recurrent AF (AUC 0.781; p<0.001).

**Conclusion:**

These findings demonstrate that visceral adiposity and metabolic alterations are independently associated with atrial dysfunction and atrial fibrillation in middle-aged individuals with cardiometabolic risk.

## Introduction

Atrial fibrillation is the most common sustained cardiac arrhythmia and a major contributor to morbidity and mortality, largely due to its association with stroke and heart failure (1). Its rising prevalence parallels the global increase in cardiometabolic disorders, particularly obesity, hypertension, and diabetes, which promote structural and electrical atrial remodeling leading to atrial cardiomyopathy (2).

Obesity-related AF is thought to be mediated in part by expansion of epicardial adipose tissue, which lies in direct contact with the atrial myocardium and shares its microcirculation. Experimental and translational models suggest that EAT can promote inflammation, fibrosis, and electrophysiological remodeling through paracrine signaling, adipokine dysregulation, and immune cell activation, thereby contributing to a vulnerable atrial substrate (3).

Middle-age is a critical period during which cardiometabolic risk factors begin to exert significant impact on cardiovascular health, often heralding the onset of complications such as AF (4). However, the complex interplay between metabolic abnormalities, subclinical organ dysfunction, and cardiac remodeling in this population remains incompletely understood.

Previous studies have predominantly focused on patients with advanced disease or overt heart failure, leading to heterogeneous cohorts that complicate the identification of independent predictors and early pathophysiological mechanisms (5). Therefore, investigating a relatively homogeneous group of middle-aged patients with cardiometabolic risk factors but without established diabetes or advanced comorbidities may provide clearer insights into the early determinants of AF onset and recurrence.

The present study aims to elucidate the conventional anthropometric, metabolic, and echocardiographic predictors associated with new-onset and recurrent AF in this selected population, with a particular focus on identifying early markers of cardiac remodeling and dysfunction that could inform preventive and therapeutic strategies in cardiometabolic medicine.

## Methods

This observational cohort study included 240 consecutive middle-aged (40–60 years) outpatients with cardiometabolic risk factors, where 169 had documented AF (1). Patients were enrolled after maintaining a stable sinus rhythm for at least 3 weeks following cardioversion. After applying stringent inclusion criteria—middle-aged, stable sinus rhythm for more than 3 weeks, preserved left ventricular (LV) ejection fraction and left ventricular global longitudinal strain (GLS), normal diastolic function and no significant valvular lesions. All eligible patients were stratified into 3 groups: 71 controls without AF, 77 with new onset AF, and 92 with recurrent AF. Obesity was defined according to body mass index, calculated as previously described, with additional calculation of body roundness index (BRI) (6).

Exclusion criteria were applied to ensure a homogeneous study population and included: older age (>60 years), coronary artery disease, valvular or congenital heart disease, any persistent arrhythmia, cardiomyopathy, LV systolic dysfunction (EF <50%), pericarditis, myocarditis, heart failure, chronic obstructive pulmonary disease, type 2 diabetes mellitus (T2DM), thyroid disease, pacemaker implantation, previous cardiac surgeries or ablation procedures, sleep apnea, anemia, neoplastic disease, alcohol abuse, chronic kidney or liver disease, and poor echocardiographic image quality.

The study population was deliberately restricted to middle-aged patients without T2DM to investigate the impact of cardiometabolic risk factors on AF development and recurrence in a relatively homogeneous cohort. Excluding patients with T2DM was necessary because this subgroup frequently exhibits extensive comorbidities and advanced laboratory and echocardiographic abnormalities, which could introduce significant heterogeneity and confound the identification of independent predictors. By focusing on a more uniform population, the study aims to elucidate early pathophysiological changes and the specific contribution of metabolic, renal, and structural cardiac alterations to AF risk, without the confounding influence of advanced disease states.

### Laboratory assessment

All patients underwent laboratory evaluation using a standard outpatient panel, including commonly assessed parameters such as blood glucose, uric acid, hsTnI, high-sensitivity C-reactive protein (hsCRP), hemoglobin, electrolytes, creatinine, and creatinine clearance. Lipid profiles were not included in the primary analysis due to the heterogeneity of the study population: some patients were receiving lipid-lowering therapy, while others had undiagnosed dyslipidemia. Including contemporary lipid measurements could therefore introduce confounding effects, obscuring true associations with atrial fibrillation. Instead, historical data regarding the presence of dyslipidemia were recorded to provide context on patients’ cardiometabolic risk profiles without affecting the main outcome analyses.

### Echocardiography

All patients underwent standard 2D echocardiography with volumetric analysis of the atria and speckle-tracking assessment. Echocardiograms were performed using a Philips EPIQ7 ultrasound system with patients in the left lateral decubitus position. Measurements were averaged over three cardiac cycles at a frame rate >50 Hz. Conventional LV structure and function parameters were assessed according to current clinical guidelines (7–9). Both left (LA) and right (RA) atria were assessed in accordance with current recommendations, with LA volumes and RA volumes and function measured using the same standardized methods (10). Electromechanical delay was measured as the time from the P-wave onset in lead II (ECG) to the peak A’-wave on the lateral wall tissue Doppler during sinus rhythm (11). Epicardial fat thickness was assessed by transthoracic echocardiography using a standard parasternal long-axis view. EAT was measured on the free wall of the right ventricle as the echo-free space between the outer wall of the myocardium and the visceral layer of the pericardium at end-diastole (12). Three consecutive cardiac cycles were recorded, and the average value was used for analysis. All measurements were performed by an experienced echocardiographer blinded to the clinical data.

### Speckle-tracking analysis

**Figure 1:**
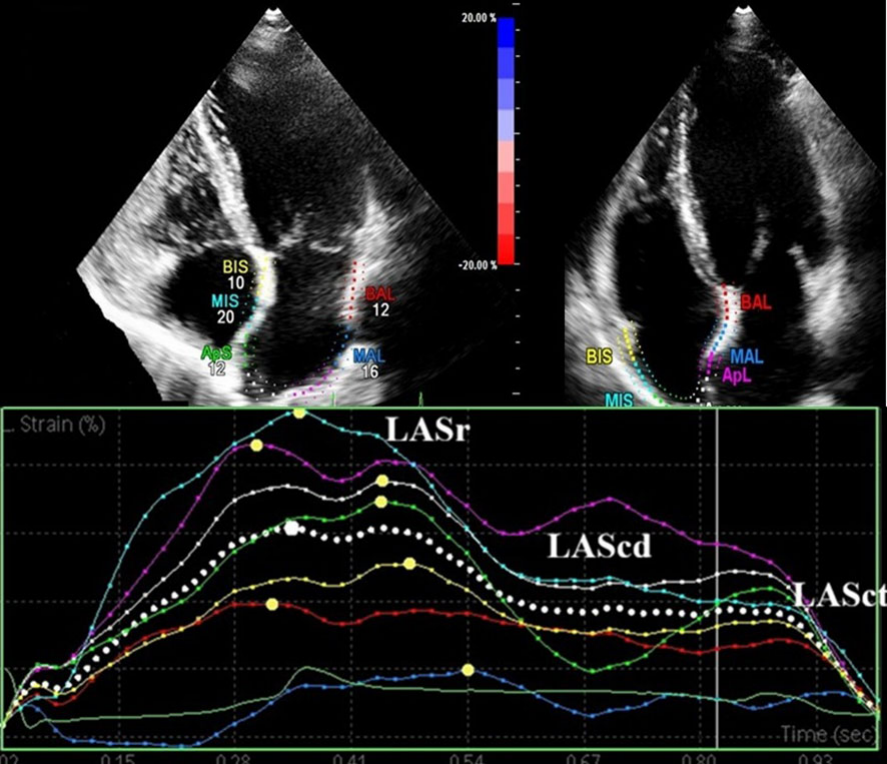
Apical four-chamber views of the left and right atria (top) and speckle-tracking–derived atrial strain curves (bottom) in a patient with atrial fibrillation.

Global longitudinal strain of the left ventricle was assessed using two-dimensional speckle-tracking echocardiography according to standard guideline-recommended methods to detect subclinical myocardial changes. LA deformation was assessed into 2 phases (10)– **Figure 1**:

Reservoir Phase (LASr %): Difference between strain at mitral valve opening and ventricular end-diastole. Contraction Phase (LASct %): Difference between strain at ventricular end-diastole and atrial contraction onset. The LA stiffness index was calculated as E/Em/LASr.

RA mechanics were assessed using the RV-focused four-chamber view. RA strain components included (10): RASr %: Strain during the reservoir phase. RASct %: Strain during the contractile phase.

#### Repeatability and reproducibility

Intra-observer variability was assessed in 40 randomly selected subjects by repeated offline analysis of the same echocardiographic loops at different time points. Agreement between measurements was evaluated using intraclass correlation coefficients (ICC). Intraclass correlation coefficients demonstrated excellent reproducibility: LASr: ICC 0.996 (95% CI 0.989–0.998; p < 0.001) and EAT: ICC 0.958 (95% CI 0.889–0.984; p < 0.001) These findings confirm high intra-observer reproducibility of strain-derived atrial parameters and echocardiographic assessment of epicardial adipose tissue in the present study.

### Statistical analysis

Data were analyzed using SPSS version 23.0. Descriptive statistics are presented as mean ± standard deviation. Categorical variables were compared using chi-square tests, with results expressed as percentages (%). Differences between independent groups were assessed using analysis of variance (ANOVA) with Bonferroni correction, after verifying the distribution of the variables and applying the necessary transformations to ensure appropriate statistical analysis. Relationships between variables were evaluated using Pearson’s or Spearman’s correlation coefficients, depending on data distribution. Binary logistic regression analysis was performed to identify independent predictors of new onset and recurrent atrial fibrillation, with odds ratios (ORs) and 95% confidence intervals (CIs) reported. Receiver operating characteristic (ROC) curve analysis was conducted to assess the discriminative ability of significant predictors, with the area under the curve (AUC) calculated to quantify diagnostic performance.

## Results

The study included 240 participants (age 40–60 years), with balanced sex distribution (119 males, 49.6%; 121 females, 50.4%). Group allocation was: controls (n=71), new-onset AF (n=77), and recurrent AF (n=92). Female sex predominated in controls (64.5%), whereas males predominated in new-onset AF (68.8%) and recurrent AF (59.8%). Dyslipidemia was present in 36% of controls, 29% of new-onset AF, and 38% of recurrent AF. Arterial hypertension was observed in 28.2% of controls (n=20), 55.8% of new-onset AF (n=43), and 57.6% of recurrent AF (n=53) (overall 48.3%, n=116).

Between-group comparisons showed significant differences in anthropometric and metabolic parameters. BMI differed across groups (28.17 ± 2.85 vs. 29.73 ± 2.84 vs. 30.18 ± 2.17 kg/m²; p=0.01). Body roundness index also differed (4.36 ± 1.30 vs. 4.51 ± 1.56 vs. 5.03 ± 1.37; p=0.04). Fasting glucose was higher in both AF groups compared with controls (5.44 ± 0.47 and 5.39 ± 0.42 vs. 5.14 ± 0.55 mmol/L; p=0.001). High-sensitivity troponin I was higher in recurrent AF (**Table 1**), hsCRP did not differ significantly between groups (p=0.198). Creatinine clearance was lower in recurrent AF (48.59 ± 3.73 mL/min; p=0.002). Uric acid levels differed between groups and were higher in AF (**Table 1**).

**Table 1:** Anthrometric and biochemical parameters.

Parameter	Controls (n=71)	New onset AF (n=77)	Reccurent AF (n=92)	p-value
BMI, kg/m2	28.17 ± 2.85	29.73 ± 2.84	30.18 ± 2.17	0.01
Body roundness index	4.36 ± 1.30	4.51 ± 1.56	5.03 ± 1.37*	0.04
Glucose, mmol/l	5.14 ± 0.55	5.44 ± 0.47	5.39 ± 0.42	0.001
hsTnI pg/ml	0.51 ± 0.55	1.23 ± 0.76	1.94 ± 1.18*	0.001
hsCRP, mg/L	1.72 ± 0.19	1.70 ± 0.15	2.11 ± 0.15	0.198
Creatinine clearance, ml/min	51.77 ± 4.22	52.28 ± 8.44	48.59 ± 3.73	0.002
Uric acid, µmol/L	273.6 ± 81.40	322.7 ± 78.32	312.59 ± 87.55	0.001

*Significant difference vs. new-onset AF group, p < 0.05. BMI, body mass index; BRI, body roundness index; hsTnI, high-sensitivity troponin I; hsCRP, high-sensitivity C-reactive protein; AF, atrial fibrillation.

Echocardiographic LV assessment showed significant between-group differences in LV mass, GLS, E/e′, and EAT (all p ≤ 0.001 as reported in **Table 2**). Right ventricular functional parameters did not differ significantly between groups (**Table 2**).

**Table 2:** Echocardiographic parameters of left ventricle.

Parameter	Controls (n=71)	New-onset AF (n=77)	Reccurent AF (n=92)	p-value
LV mass, gr/m2	55.62 ± 10.60	78.28 ± 15.24	79.88 ± 11.90	0.001
GLS, %	-21.93 ± 1.15	-20.73 ± 0.94	-20.75 ± 0.94	0.001
E/E’	7.13 ± 0.86	8.9 ± 1.93	9.84 ± 3.31*	0.001
ЕАТ, mm	3.79 ± 0.56	6.20 ± 1.86	6.14 ± 1.73	0.001

*Significant difference vs. new-onset AF group, p < 0.05. LV mass, left ventricular mass indexed to body surface area; GLS, global longitudinal strain of left ventricle; E/E’, ratio of early diastolic mitral inflow velocity to early diastolic mitral annular velocity; EAT, epicardial adipose tissue thickness; AF, atrial fibrillation.

**Figure 2:**
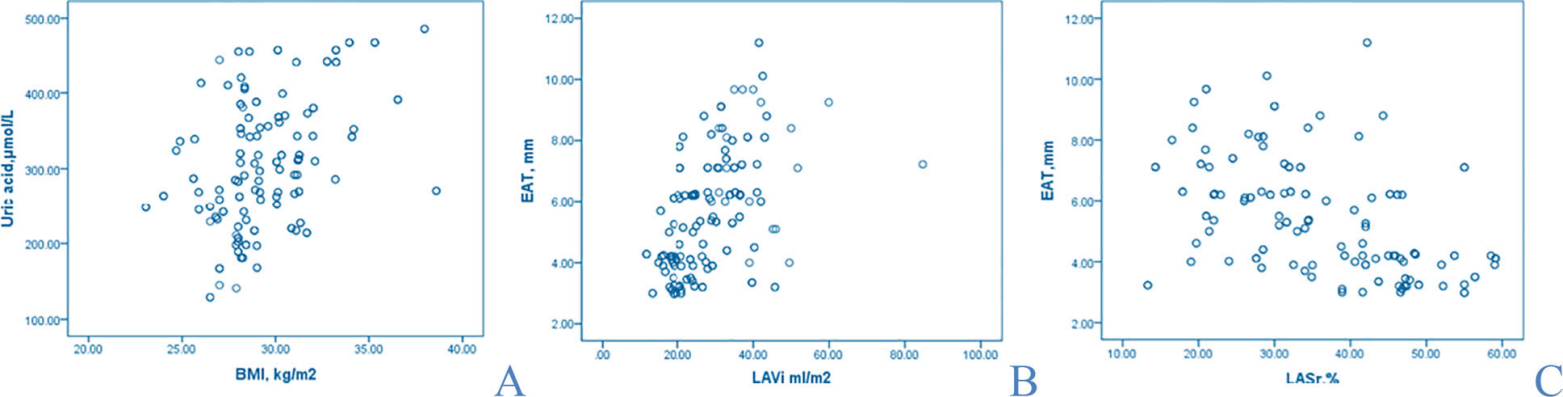
Correlation analysis between uric acid and BMI **(A)**, EAT and LAVi **(B)**, EAT and LASr **(C)**.

Atrial remodeling was reflected by differences in LAVi and LATEF (**Table 3**). LA reservoir strain was lower in new-onset and recurrent AF compared with controls (47.50 ± 6.00 vs. 33.60 ± 10.29 vs. 29.20 ± 8.99%; p=0.001). LA contractile strain also differed (−19.10 ± 7.00 vs. −14.50 ± 6.66 vs. −14.12 ± 6.80%; p=0.001). LA stiffness differed between groups (0.15 ± 0.02 vs. 0.37 ± 0.16; p=0.001). Electromechanical delay was prolonged in AF groups and mechanical dispersion was higher in recurrent AF (**Table 3**). Uric acid correlated with BMI (r=0.410; p<0.001) (**Figure 2A**). EAT correlated with BMI (r=0.352; p<0.001), negatively with LASr (r=−0.481; p<0.001) (**Figure 2C**), and positively with LAVi (r=0.527; p<0.001) (**Figure 2B**).

**Table 3:** Echocardiographic parameters of left and right atrium.

Parameter	Healthy (n=71)	New onset AF (n=77)	Reccurent AF (n=92)	p-value
LAVi, ml/m²	21.15 ± 7.06	28.83 ± 8.86	31.16 ± 9.81	0.001
LATEF, %	71.96 ± 8.27	64.90 ± 11.30	59.04 ± 14.90	0.001
LASr, %	47.50 ± 6.00	33.60 ± 10.29	29.20 ± 8.99*	0.001
LASct, %	-19.10 ± 7.00	-14.50 ± 6.66	-14.12 ± 6.80	0.001
LA stiffness	0.15 ± 0.02	0.29 ± 0.11	0.37 ± 0.161*	0.001
EMD, ms	30.97 ± 20.20	46.36 ± 21.15	40.05 ± 18.10	0.001
MD, ms	38.00 ± 40.40	48.42 ± 43.53	82.05 ± 68.72*	0.001
RAVi, ml/m²	16.00 ± 6.78	24.36 ± 8.70	22.70 ± 7.78	0.001
RATEF, %	57.64 ± 10.74	56.17 ± 12.44	53.06 ± 16.40	0.091
RASr, %	37.79 ± 10.60	32.06 ± 9.77	28.43 ± 9.15	0.001
RASct, %	-16.38 ± 7.79	-12.88 ± 5.16	-12.83 ± 5.50	0.001
RA stiffness	0.11 ± 0.08	0.15 ± 0.60	0.16 ± 0.07	0.001

LAVi, left atrial volume index; LATEF, left atrial total emptying fraction; LASr, left atrial reservoir strain; LASct, left atrial contractile strain; LA stiffness, left atrial stiffness index; EMD, electromechanical delay; MD, mechanical dispersion; RAVi, right atrial volume index; RATEF, right atrial total emptying fraction; RASr, right atrial reservoir strain; RASct, right atrial contractile strain; RA stiffness, right atrial stiffness index; AF, atrial fibrillation.

In multivariable logistic regression, independent predictors of new-onset AF were fasting glucose (OR 3.604; 95% CI 1.338–9.704; p=0.011), EAT (OR 1.479 per 1 mm; 95% CI 1.121–1.950; p=0.006), electromechanical delay (OR 1.043; 95% CI 1.017–1.071; p=0.001), uric acid (OR 1.006; 95% CI 1.001–1.012; p=0.026), and LASr (OR 0.944; 95% CI 0.893–0.999; p=0.045).

Independent predictors of recurrent AF were age (OR 1.122; 95% CI 1.054–1.193; p<0.001), BMI (OR 1.209; 95% CI 1.020–1.433; p=0.028), hsTnI (OR 3.546; 95% CI 2.177–5.776; p<0.001), and LASr (OR 0.845; 95% CI 0.778–0.917; p<0.001).

**Figure 3:**
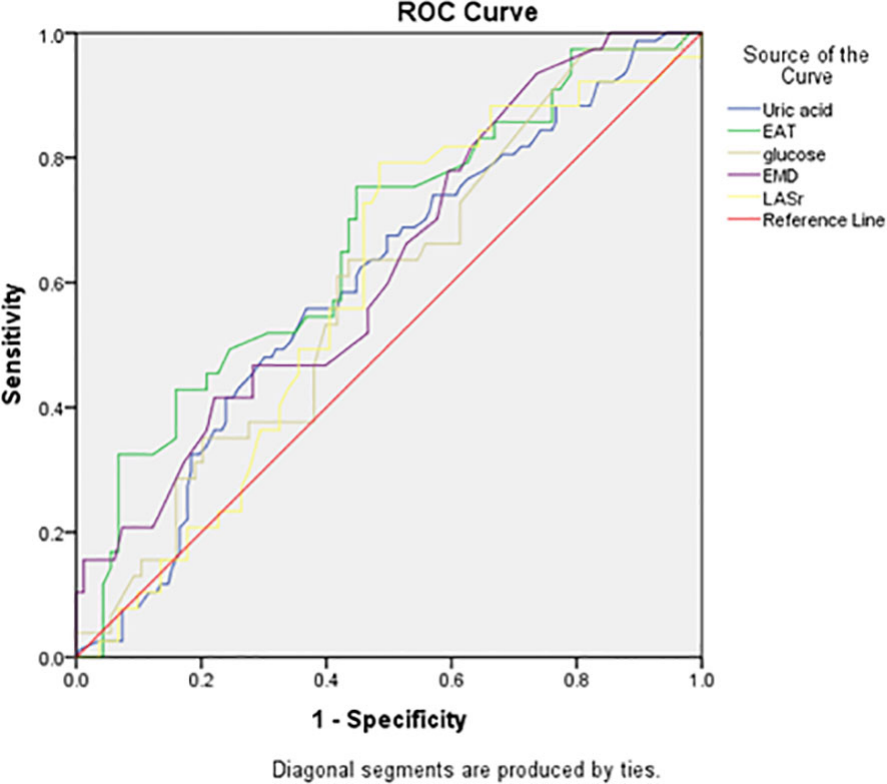
ROC curve analysis of the identified predictors for new- onset AF.

For new-onset AF, EAT showed the highest discrimination (AUC 0.664; 95% CI 0.591–0.738; p<0.001), followed by mechanical dispersion (AUC 0.628; 95% CI 0.555–0.702; p=0.001). Uric acid (AUC 0.599; p=0.013), glucose (AUC 0.591; p=0.023), and LASr (AUC 0.591; p=0.023) showed modest discrimination **Figure 3**.

**Figure 4:**
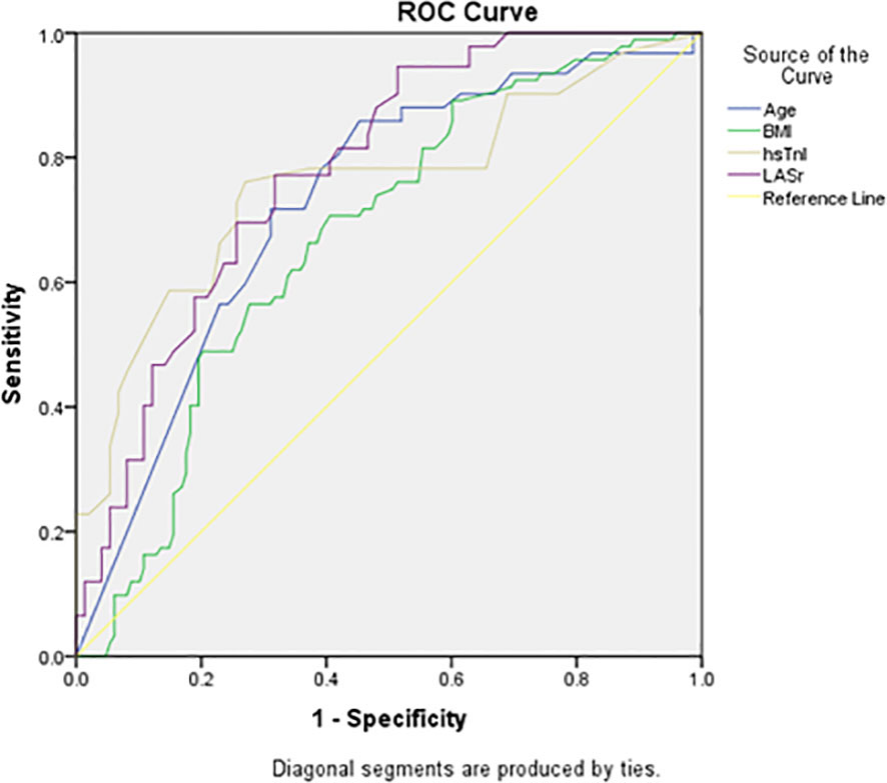
ROC curve analysis of the identified predictors for recurrent AF.

For recurrent AF, LASr had the highest discrimination (AUC 0.781; 95% CI 0.724–0.838; p<0.001), followed by hsTnI (AUC 0.758; 95% CI 0.692–0.825; p<0.001), age (AUC 0.729; 95% CI 0.664–0.794; p<0.001), and BMI (AUC 0.673; 95% CI 0.604–0.741; p<0.001) **Figure 4**.

## Discussion

The main findings of our study are that both new onset and recurrent AF in middle-aged patients are associated with distinct metabolic, structural, and functional alterations, which appear to be associated with early stages of disease onset and recurrence. Importantly, our results highlight the limitations of traditional anthropometric measures such as BMI in assessing cardiometabolic and arrhythmic risk. Although direct imaging-based quantification of total visceral fat was not performed, patients with new-onset AF, despite not meeting conventional BMI criteria for obesity, exhibited significantly increased visceral adiposity, as reflected by BRI and EAT. This underscores the importance of evaluating fat distribution rather than relying solely on BMI, particularly in patients with subtle metabolic derangements (6).

Obesity is a chronic and staged disease with a progressive nature, leading to a spectrum of cardiometabolic complications. Both hypertension and AF are recognized as obesity-related cardiovascular disorders, arising through shared mechanisms of hemodynamic overload, neurohormonal activation, and atrial and ventricular structural remodeling (2–6). Renal impairment further compounds the risk in obese patients, potentially progressing to cardiometabolic-renal (CMR) syndrome. CMR syndrome is a systemic disorder characterized by complex interactions among metabolic risk factors, chronic kidney disease (CKD), and the cardiovascular system, resulting in multiorgan dysfunction and a high rate of adverse cardiovascular outcomes (13). Recognizing these staged interactions creates opportunities for early intervention, potentially halting progression and preventing obesity-related cardiometabolic complications.

Our findings underscore the high burden of cardiometabolic risk in middle-aged obese outpatients, reflecting the complex interplay of metabolic, cardiovascular, and renal factors that characterizes CMR syndrome (13). Dyslipidemia was observed in 29–38% of patients across AF groups and controls, while hypertension affected over half of patients with new onset or recurrent AF, highlighting the frequent coexistence of modifiable cardiovascular risk factors. The presence of modestly reduced creatinine clearance in all groups suggests early renal impairment, which, in the context of obesity and cardiometabolic risk, likely represents the initial stages of chronic kidney disease (13). Although albuminuria and proteinuria were not assessed, these findings support the concept of a cardiometabolic-renal continuum, wherein metabolic risk factors, renal dysfunction, and cardiovascular pathology interact to amplify the risk of adverse outcomes.

In our cohort, metabolic alterations were accompanied by changes in atrial and ventricular geometry and function, reflecting the combined consequences of obesity, hypertension, and chronic kidney disease. These interrelated disturbances highlight the early substrate for atrial fibrillation, with both structural remodeling and functional impairment predisposing to arrhythmia onset and recurrence (1–6).

Metabolic derangements were evident, as fasting glucose and uric acid levels were elevated in AF patients, and high-sensitivity troponin I was significantly higher in recurrent AF, suggesting the presence of low-grade myocardial injury (14, 15). The association between hsTnI and recurrent AF is consistent with previous reports indicating that high-sensitivity troponins reflect ongoing subclinical myocardial stress and are linked to adverse outcomes in AF populations. Although overt structural heart disease was excluded in our cohort, the observed relationship with recurrence may indicate subtle myocardial involvement that persists despite preserved left ventricular function (14, 15). Elevated uric acid levels are associated with a higher risk of developing AF, even in individuals without established cardiovascular disease or traditional risk factors. The proarrhythmic effect of hyperuricaemia may arise from direct mechanisms including oxidative stress, inflammation, and ion-channel modulation, as well as indirect influences reflecting underlying cardiometabolic conditions such as hypertension, chronic kidney disease, obesity, and diabetes (16). Notably, the association appears particularly strong in patients with cardiometabolic disorders. Elevated uric acid has been identified as an independent predictor of new-onset AF, and further studies are warranted to determine whether uric-acid–lowering therapies could reduce AF risk (16).

Echocardiographic assessment revealed marked structural and functional remodeling. Left ventricular mass and global longitudinal strain indicated subclinical LV remodeling and systolic impairment. The E/e′ ratio progressively increased across groups, although values remained below the threshold for elevated filling pressures (17). Epicardial adipose tissue was significantly greater in AF patients, reinforcing the link between visceral fat and atrial remodeling (3, 18).

Our findings align with prior evidence demonstrating an association between epicardial adipose tissue and AF presence and severity, supporting the concept that visceral fat depots contribute to atrial remodeling beyond the information captured by body mass index (2, 3, 18). Previous studies have reported associations between increased EAT and atrial enlargement, impaired atrial function, and AF burden, consistent with the observed correlations between EAT, left atrial volume index, and left atrial reservoir strain in our cohort. The moderate discriminatory performance of EAT for new-onset AF further suggests that EAT should be interpreted primarily as an early pathophysiological marker rather than a standalone diagnostic or screening tool.

Atrial structural and functional parameters demonstrated prominent alterations: both left and right atrial volumes were increased, atrial reservoir and contractile strains were reduced, and atrial stiffness was markedly elevated in recurrent AF. Electromechanical abnormalities, including prolonged atrial electromechanical delay and increased mechanical dispersion, further highlighted the propensity for arrhythmogenesis. Collectively, these findings point to a coordinated pattern of metabolic, structural, and functional derangements that create a substrate for both new-onset and recurrent AF, highlighting the potential value of early risk stratification and targeted preventive strategies (19–26).

Functional atrial remodeling also emerges as a key determinant. Reduced LASr was independently associated with both new-onset and recurrent AF, underscoring the importance of impaired atrial mechanical function (27–32). However, ROC analyses demonstrated only modest discrimination for predictors of new-onset AF (AUC range 0.59–0.66). These markers should therefore be interpreted primarily as indicators of early pathophysiological remodeling rather than standalone diagnostic or screening tools. Their clinical relevance may lie in multiparametric risk assessment rather than individual predictive performance. Although patients with AF do not typically present with LASr values <23% a threshold that formally defines atrial cardiomyopathy, this nevertheless indicates that, in practice, these patients are already at increased risk for developing such atrial disease (33). Interestingly, age and BMI were associated with recurrent AF but did not independently predict new onset AF in this cohort, suggesting that cumulative exposure to metabolic and structural stressors may contribute over time, predisposing to recurrence and other cardiometabolic complications (34).

While our study did not evaluate lifestyle interventions, existing evidence supports the role of Mediterranean dietary patterns in improving visceral adiposity, insulin sensitivity, and systemic inflammation, pathways that are implicated in atrial remodeling (35, 36).

### Study limitations

This study has several limitations. First, its observational cohort design precludes causal inference between identified risk factors and AF development or recurrence. Second, follow-up was limited to a single telephone contact at the 5-year time point, restricting detection of interim cardiovascular events or changes in AF status. Third, advanced metabolic markers, including fasting insulin and glycated hemoglobin (HbA1c), were not measured, limiting mechanistic insights. Fourth, renal function was assessed only by creatinine clearance (estimated glomerular filtration rate), without additional measures such as albuminuria or cystatin C, potentially underestimating early kidney impairment. Fifth, lipid profiling was not systematically evaluated due to population heterogeneity and widespread use of lipid-lowering therapies. Sixth, echocardiographic assessment of epicardial adipose tissue was performed with standard echocardiography rather than gold-standard imaging modalities such as CT or MRI, which may have provided more precise quantification. In addition, body composition was assessed using conventional anthropometric indices rather than direct quantification of visceral fat by imaging techniques such as DEXA or abdominal ultrasonography. Finally, the relatively modest sample size may limit statistical power, increase the risk of model overfitting in multivariable analyses, and reduce generalizability. The cohort consisted of middle-aged outpatients with cardiometabolic risk factors, further restricting external applicability.

Accordingly, these findings should be regarded as exploratory and hypothesis-generating. Prospective validation in larger, independent cohorts is required before translation into routine clinical practice.

## Conclusion

Our study underscores the value of a multiparametric approach in assessing middle-aged obese patients with cardiometabolic risk. Integrating metabolic markers with structural and functional cardiac indices may facilitate earlier identification of individuals at risk for both new onset and recurrent AF. These findings support the concept of a cardiometabolic continuum, in which metabolic disturbances, cardiac remodeling, and early renal impairment interact to amplify the risk of adverse outcomes. Early recognition of high-risk patients through multiparametric profiling may inform targeted preventive interventions, including glycemic control, blood pressure management, weight reduction, and close cardiac monitoring.

## Data Availability

The raw data supporting the conclusions of this article will be made available by the authors, without undue reservation.
